# Critical role of calnexin in Nef-mediated inactivation of ABCA1

**DOI:** 10.1186/1742-4690-8-S2-P8

**Published:** 2011-10-03

**Authors:** Lucas Jennelle, Michael Bukrinsky

**Affiliations:** 1 Department of Microbiology, Immunology and Tropica I Medicine, The George Washington University, Washington, DC 20037, USA

## 

Our previous studies demonstrated that HIV-1 Nef impairs the function of the main cellular cholesterol transporter, ABCA1, thus affecting reverse cholesterol transport, the key anti-atherogenic mechanism of the body. Nef has been shown to interact with ABCA1 through a DDDHLK motif in the C-terminal cytoplasmic domain, but, surprisingly, this interaction was not essential for ABCA1 inactivation suggesting that Nef may downregulate ABCA1 through an effect on an intermediate protein. We used mass spectrometry to search for ABCA1 interacting proteins whose binding to ABCA1 was altered by Nef. This search identified calnexin, a transmembarne protein that functions as an endoplasmic reticulum chaperone regulating maturation of glycosylated proteins. Calnexin, but not another endoplasmic reticulum chaperone Calreticulin, co-immunoprecipitated and co-localized with ABCA1 in the absence of Nef, and this interaction was disrupted by wild-type, but not by non-myristoylated Nef (Nef G2A), suggesting that membrane association was critical for this effect of Nef. Interestingly, Nef also interacted with calnexin, despite not being glycosylated, and this interaction was mapped to the region between amino acid residues 45 and 65 of Nef. Although calnexin has not been implicated in ABCA1 maturation, it is known to regulate maturation of other ABC transporters. Consistent with this notion, siRNA-mediated inactivation of calnexin resulted in surface expression of non-functional ABCA1 with shortened half-life, reproducing the effects of Nef. ABCA1 inactivation by Nef was reversed by lysosomal inhibitor chloroquine, suggesting involvement of lysosomes in Nef-dependent ABCA1 degradation. The DDDHLK→AAAAAA mutant of ABCA1, which showed greatly reduced binding to Nef, interacted with calnexin and this interaction was abrogated in the presence of Nef. These results suggest that Nef inactivates ABCA1 by blocking the interaction between this cholesterol transporter and calnexin, the cellular endoplasmic reticulum chaperone involved in regulation of folding and maturation of glycosylated proteins. Our results are consistent with a model whereby Nef disrupts the interaction between calnexin and ABCA1 by a competitive mechanism, via binding to calnexin and preventing its interaction with ABCA1, thus affecting ABCA1 maturation and promoting its degradation via the lysosomal pathway.

